# International organizations and migrant health in Europe

**DOI:** 10.1186/s40985-016-0033-4

**Published:** 2016-10-20

**Authors:** Alexander E. Kentikelenis, Amanda Shriwise

**Affiliations:** 1 Department of Sociology, University of Oxford, Oxford, UK; 2 Department of Social Policy and Intervention, University of Oxford, Oxford, UK; 3 Trinity College, Broad Street, Oxford, OX1 3BH UK

**Keywords:** International organizations, Migrant health, Migration, Refugees, Public health

## Abstract

International organizations have defined and managed different aspects of migrant health issues for decades, yet we lack a systematic understanding of how they reach decisions and what they do on the ground. The present article seeks to clarify the state of knowledge on the relationship between international organizations and migrant health in Europe. To do so, we review the operations of six organizations widely recognized as key actors in the field of migrant health: the European Commission, the Regional Office for Europe of the World Health Organization, the International Organization on Migration, Médecins du Monde, Médecins Sans Frontières, and the Open Society Foundation. We find that international organizations operate in a complementary fashion, with each taking on a unique role in migrant health provision. States often rely on international organizations as policy advisors or sub-contractors for interventions, especially in the case of emergencies. These linkages yield a complex web of relationships, which can vary depending on the country under consideration or the health policy issue in question.

## Background

In 2015, Europe received the largest inflow of refugees and asylum seekers since World War II, fleeing conflicts in Syria, Afghanistan, Iraq, and elsewhere. The International Organization for Migration (IOM) estimates that 1,046,600 migrants arrived in Europe by land and sea in 2015—nearly four times as many as in 2014 [1]. This situation reportedly “overwhelmed” [2] national authorities and calls for international solutions quickly ensued. International non-governmental organizations were quick to deploy missions at key points along migratory routes, and intergovernmental organizations supported national and regional policies and also designed interventions on the ground. At the same time, under the auspices of the European Union (EU), the continent’s leaders met to discuss possible responses to the increased migratory flows, to assign responsibility for the provision of basic services, and to decide on a process for relocating refugees and asylum seekers across the EU’s 28 member states. Nevertheless, despite repeated summits and policy declarations, the implementation of agreed actions remained elusive, and—at the time of writing—the Union continues to grapple with how best to respond to the influx of migrants.

As this experience suggests, international organizations have been central to defining and handling different aspects of the migrant “crisis” in Europe—from macro-level policy design to micro-level interventions. This should come as no surprise: international actors have been involved in migration issues for decades [3–5]. As early as 1983, the World Health Regional Office for Europe (WHO EURO) held its first conference on migrant health in the Netherlands [3–6], and since then, international organizations have been active in multiple ways to ensure the right to health for non-nationals, including migrants and refugees.

However, despite their importance, we lack a systematic understanding of what motivates international organizations, how they reach decisions, and what they do on the ground. This article seeks to clarify the relationship between international organizations and migrant health in Europe, an issue that has received little attention in academic literature. Based on our findings, we also outline promising directions for future research.

The article is structured as follows. The “International organizations in the world system” section presents an overview of the general social science debates over international organizations and discusses key aspects of their activities. The “Methods” section outlines the methodological strategy we employed to identify how international organizations operate in the field of migrant health in Europe. The “Findings” section presents our findings for six major intergovernmental or international non-governmental organizations. We conclude by discussing the implications of our findings and proposing avenues for future work.

## International organizations in the world system

In a globalized environment, states increasingly resort to supranational solutions to address policy problems, and—correspondingly—international organizations have grown in number, size, and importance [7–11]. At the most general level, these organizations serve as purposive actors that shape debates, carry out interventions, and make rules that are then diffused around the world [5, 12–15].

Yet, international organizations are also a heterogeneous group, commanding different material and ideational resources. A fundamental distinction is between the intergovernmental or non-governmental nature of these organizations. Intergovernmental organizations (IGOs) former are created and controlled by states and—as a result—carry considerable authority; they are generally the first port of call when states wish to address an international policy problem. For example, the World Health Organization has the authority to shape how we understand the determinants of health outcomes [16]. In contrast, international non-governmental organizations’ (INGOs) membership is commonly composed of individuals or other private or non-governmental organizations. Both legal personality (governmental or non-governmental) and resources affect how these organizations respond to policy problems. Here, we present a brief overview of social scientific research into three aspects of international organizations—goals and mandates, governance and financing, and outputs—that set the stage for our review.

### Organizational goals and mandates

Organizations come into being as a result of purposive action, and their mandates reflect the policy preferences of their founders [17–19]. In the case of IGOs, the founders are commonly states that wish to establish organizational frameworks to address coordination challenges [10, 11]. The outcomes are IGOs with rigid mandates—codified in founding treaties—that define their policy remits, available resources, and enforcement mechanisms. For example, UN peacekeeping missions are required to appear impartial, which can constrain their ability to intervene in conflicts and humanitarian crises—even when such crises fall within their remit and when resources to intervene are available [20, 21].

The geopolitical circumstances imprinted on these mandates have important implications for organizational behavior because they affect what is considered to be permissible policy action. They also create strong inertial forces that can make organizational change and adaption difficult. A salient example of these processes in the field of international migration relates to the division of labor between the IOM and the United Nations High Commissioner for Refugees (UNHCR): the former holding a mandate on “voluntary” migration and the latter on “forced” migration. While the IOM has gradually expanded its remit to cover some issues of forced migration (albeit with no legal protection mandate), the UNHCR has upheld the forced-voluntary migration distinction [22, 23], even though the boundaries between the two are often unclear [24].

For their part, INGOs can be understood as “authoritative transnational bodies employing limited resources to make rules, set standards, propagate principles, and broadly represent ‘humanity’ vis-à-vis states and other actors” [25]. They do so in various ways (examined below), but a crucial difference from IGOs is their relative flexibility: how they define their goals or amend their mandates is not primarily a function of global politics (as in IGOs) but a reflection of the preferences of their management, available resources, evolving policy knowledge, and intra-organizational dynamics.

### Governance and financing

As suggested above, IGOs’ authority is conferred by states. To ensure that IGOs fulfill but do not transgress their mandate, member states have put in place oversight mechanisms, akin to boards of directors of companies [11]. These structures—commonly called Executive Boards—have varying degrees of power over organizational decision-making. In some cases, they meet multiple times per week to manage day-to-day activities (e.g., in the World Bank); in other cases, they only convene a few times per year to provide broad guidance (e.g., in the World Health Organization). Of course, states can also express satisfaction or disapproval with organizational policies via the power of the purse. Most IGOs depend on contributions from their member states in order to employ staff and to develop programs and interventions. This makes IGOs sensitive to the preferences of their most powerful constituents for fear of alienating them and thereby losing resources and relevance [14]. For example, the US “tamed” the Inter-American Development Bank, when the organization chose to disregard US views over the appropriate policy content of its interventions for a brief period in the 1980s [26].

In contrast, governance mechanisms and financing differ considerably across INGOs, thereby precluding easy comparisons. Large INGOs generally share features resembling those of IGOs or private companies, with boards of directors separate from management, defined codes of conduct, and organizational chains of command [27]. The financing of INGOs can emanate from states, IGOs, other INGOs, the private sector, the public at large, or some combination of these. While this allows INGOs to exhibit creativity in fundraising, it also makes them sensitive to reputational challenges: if an INGO primarily depends on individual donations, then allegations of misconduct or mismanagement of funds could affect the public’s willingness to donate, which in turn may threaten the survival of the organization. For instance, recent allegations of corruption at Save the Children, a prominent INGO, yielded a barrage of negative press coverage and denouncements [28].

### Organizational outputs

International organizations are more than institutionalized arenas for deliberation; their power also emanates from their capacity to act. We distinguish between two types of outputs. First, both IGOs and INGOs commonly deploy interventions within countries. These interventions can take place at the macro- or micro-levels, that is, influencing policy design at the national or regional level or deploying programs that operate locally. For example, the WHO often provides advice and technical support to its members on how to organize or reform their health systems, dispatches missions to assist policy design by country officials, or prepares country reports and research notes that aid policymaking, as well as developing ad hoc micro-level interventions, like immunization programs in crisis zones [29].

Second, international organizations also have considerable ideational power by fixing meanings, diffusing norms, and collecting data. Refugees provide a case in point. Drawing on its mandate and expertise, the UNHCR was able to redefine who was to be considered a refugee under international law, ultimately transforming an entire field of policy interventions [5]. In policy environments marked by high degrees of uncertainty, this meaning-granting function can be a significant source of power for international organizations [30].

## Methods

In this paper, our main goal is to review the activities of international organizations—both IGOs and INGOs—in the field of migrant health in Europe, a research topic that has received little scholarly attention. By “Europe,” we refer to countries in the EU or European Free Trade Association (EFTA): Austria, Belgium, Bulgaria, Croatia, Cyprus, Czech Republic, Denmark, Estonia, Finland, France, Germany, Greece, Hungary, Iceland, Ireland, Italy, Latvia, Liechtenstein, Lithuania, Luxembourg, Malta, the Netherlands, Norway, Poland, Portugal, Romania, Slovakia, Slovenia, Spain, Sweden, Switzerland, and the UK. Migrants are defined as the foreign-born population, whose country of origin lies outside the EU/EFTA; in other words, we do not consider intra-EU/EFTA population movements.

### Case selection

Social scientific knowledge is best advanced by the comparative study of “crucial” cases, understood as cases marked by “their heightened importance in the intersubjective world of the community of scholars” [31]. Correspondingly, we review the operations of six international organizations that are widely acknowledged as central actors in migrant health in Europe [32–34]. Among IGOs, we focus on the European Commission (EC), the IOM, and the WHO. While other IGOs also have a remit in related areas—for example, the UNHCR—the three selected organizations have operations that relate to *both* migration and health as a core part of their operations. Among INGOs, we focus on Médecins du Monde (MdM), Médecins Sans Frontières (MSF), and the Open Society Foundations (OSF). These INGOs were selected for the breadth of their activities, including policy advocacy, ground-level operations, grant-making capacity, and overall mandate vis-à-vis migrants. Other INGOs—like the International Red Cross or the International Medical Corps—also have wide-ranging involvement in this policy area, yet space considerations did not permit a review of their activities and future research can take on this task.

### Search strategy

We developed a three-pronged search strategy. First, we searched the websites of the six organizations under review, as well as secondary literature, for information on their mandate, governance, and activities in the field of migrant health in Europe. Second, we searched two electronic databases (PubMed/MEDLINE and Web of Science) for academic literature. The database search was conducted in January 2016 using various combinations of key words for the three main axes of interest: international organizations, migrant health, and the study setting (EU/EEA). The search terms included the name of each organization reviewed, migration-related terms (“migration,” “immigration,” “migrant*,” “immigrant*,” “asylum seeker*,” “refugee*”), and study-setting terms (“Europe*” and the names of EU/EEA countries). Third, we searched for gray literature on the topic using Google and Google Scholar, using a similar methodology to the database search outlined above.

### Search results

Reflecting the dearth of empirical research on the topic, our academic literature searches yielded few relevant results: six articles on the EC [33–38], one on the WHO [39], three on the IOM [40–42], two on MdM [43, 44], two on MSF [45, 46], and none on the OSF. Our criterion for relevance was reference to international organizations’ interventions related to migrant health in Europe. We present our findings predominantly on the basis of information provided by the organizations themselves and gray literature, as well as the academic publications identified via the comprehensive review. Given that systematic evidence is unavailable, the results were achieved through narrative synthesis or the qualitative fusion of evidence and findings from multiple sources to generate new insights in a way that is both systematic and transparent [47]. This approach also aids identifying questions and hypotheses for future research, an issue to which we return in the concluding section.

## Findings

### European Commission (EC)

#### Background and governance

Founded in 1958, the EC is the executive body of the EU and is responsible for proposing legislation, enforcing European law, setting policy objective and priorities, overseeing the EU budget and policy implementation, and representing the EU outside of Europe [48]. Currently, the EC is composed of 28 Commissioners (one from each member state) and meets weekly and also on an as-needed basis in the event of major political events and crises. The EC has a bureaucracy of approximately 23,000 working in a number of the so-called Directorates-General (DGs). The DGs draft legislation and make recommendations to the Commissioners, manage funding, and coordinate public consultation related to EU initiatives [48]. The DGs related to migrant health include Health and Food Safety (SANTE), with a mandate to protect and improve public health [49]; Migration and Home Affairs (HOME), which is charged with the development of a “balanced and comprehensive EU migration policy, based on solidarity and responsibility” [50] and is working on the creation of a Common European Asylum System [50]; and Humanitarian Aid and Civil Protection (ECHO), with a key remit to assist in emergencies outside the EU [51].

The EC is financed by contributions from its member states. In 2014, the EU budget was €143 billion or around 1 % of EU gross national income [52]. In particular, SANTE oversees both public health and the European Centre for Disease Prevention and Control, with an operating budget of €496 million in 2014. HOME manages the Asylum, Migration and Integration Fund, which was created to “promote the efficient management of migration flows and the implementation, strengthening and development of a common Union approach to asylum and immigration” [53]. The Fund was allocated €3.1 billion for 2014–2020 and is committed to achieving four objectives: strengthening and developing the Common European Asylum System, supporting legal migration to the EU in line with labor demands, enhancing fair and effective return strategies, and ensuring solidarity between EU States by ensuring that EU States most affected receive adequate support. [53] ECHO’s 2014 budget came to €1.27 billion, most of which was directed to programs in the Middle East and Mediterranean (€372 million) and Africa (€572 million).

#### Migrant health-related activities in Europe

The EC’s activities related to migrant health are multifaceted, even though the regulation and provision of health care services to migrants is a national competence. The EC provides financing, usually in the form of grants, to both IGOs (such as the IOM—see below) and INGOs (such as MSF—see below) working to improve migrant health in line with EU policy. It also works to promote the instillation of minimum standards for access to health care for asylum seekers across member states in order to promote compliance with European and international law [54]. The EC’s minimum standards for health care for asylum seekers include access to emergency care, essential treatment of illness, and necessary medical or other assistance to those with special needs [6].

The EC’s DGs also finance and undertake a number of activities related to migrant health, oftentimes in partnership with the organizations discussed below [55, 56]. DG SANTE prepares and disseminates training materials to health professionals to improve the accessibility of health services for migrants in consultation with other IGOs, such as the IOM, and national governments [57, 58]. It has also funded “Equi-Health,” an IOM project (see below) to improve and tailor health care services for migrants [59], and also “MIGHEALTHNET,” an information network on good practice in health care for migrants and minorities [60–62]. Through its management of funds such as the Asylum, Migration and Integration Fund, DG HOME has supported intercultural mediation between migrants and medical staff to prevent misunderstandings and promote non-discriminatory access to health care [63] and has also provided funds to INGOs and other third sector organizations to provide and develop mental health services for asylum seekers suffering from mental health problems. [64, 65] DG ECHO provides humanitarian aid—including food, water, basic hygiene, and health care—to individuals in sending and transit countries [66] and implements EU Civil Protection Mechanisms [67] in sending countries in times of crisis, including the deployment of newly formed specially trained medical and public health teams, known as the European Medical Corps, to provide emergency medical assistance [68, 69].

### International Organization for Migration (IOM)

#### Background and governance

Founded in 1951, the IOM is currently composed of 162 member states and a large number of non-state observers, including the EU (see above), the WHO (see below), and many INGOs [70]. The organization has a mandate to provide technical and operational support for “orderly migration” [71]. The IOM founding treaty promotes a vision of a networked organization that cooperates with other IGOs, governments, and NGOs and acts as a forum for international policy coordination [71].

The organization’s highest decision-making body is its Council that convenes annually, where all member states are represented on a one-country-one-vote basis. The Council is aided by a sub-committee on programs and finance, which convenes biannually and considers the organization’s activities and financing in greater depth. The Council elects the Director General, who heads the organization’s bureaucracy and has wide authorities, as envisaged by the mandate [71]. The organization’s remit supports the provision of health assessments to migrants [72], and its bureaucracy includes a dedicated Migration Health Division, with a mission to promote “partnerships, networks and multi-country frameworks that ensure migrants’ improved physical, mental and social well-being” [72]. This IOM department has organized key policy-shaping activities around the world, like the first global consultation on migrant health [73].

The financing of IOM emanates from its member states and sponsors, like the EU, United Nations agencies, or voluntary agencies. In 2015, the organization managed an operational budget of $856.9 million [72], reflecting an increase of 14 % compared to 2014. Of these funds, the Migration Health Division absorbed 10.4 %, a figure expected to rise to 12.7 % in 2016. The majority of this Division’s funds are expended in Africa and Asia; its activities in Europe absorbed $9.7 million in 2015 [72].

#### Migrant health-related activities in Europe

The IOM is involved in migrant health activities in a variety of ways. First, the majority of the IOM’s European region migrant health budget is devoted to conducting health assessments and providing travel health assistance ($7.9 million in 2015) [74]. These assessments are conducted by physicians with the aim of collecting migrants’ medical and immunization history, conducting infectious disease screening, proving treatment or referral to local health authorities, and assessing fitness to travel [75]. For instance, the organization provides a tuberculosis detection program for UK visa applicants from 41 countries [76].

Second, the Migration Health Division of the IOM’s European office administered a major program aiming at health promotion and improved access to health services. The Equi-Health project (2013–2015), co-financed by the EC, included dedicated action on migrant health in the EU’s southern borders (Bulgaria, Croatia, Greece, Italy, Malta, Spain, and Portugal) that entailed health assessments, training of health service professionals, data collection, and capacity building in public health authorities, as well as several activities on the structural issues related to migrant health, including information collection of national legal frameworks and the development of guidelines on access to health care services [59]. In addition, the organization developed public health guidelines related to border management and the operation of detention centers [77].

Further, the IOM has been active in agenda-setting activities. For instance, in recent years, the organization has launched consultations—with the support of the EU or European governments—on migrant health issues [78].

In 2015, the IOM assumed a central role in the policy response to the refugee crisis in Europe. First, the organization collects and publishes data on the numbers of refugees and other migrants arriving in Europe on a daily basis and their migratory routes into and within the continent (including bottlenecks as a result of closed borders or other impediments to mobility) [79]. Second, the IOM head office in Geneva collects data on deceased or missing migrants on their route into Europe, an initial step in developing comprehensive policy for avoiding unwarranted deaths [80]. Finally, the organization has deployed teams in countries of entry to provide health services to incoming migrants and support the national governments with the provision of technical expertise [81, 82].

### World Health Organization (WHO)

#### Background and governance

Founded in 1948, the WHO consists of 194 member states and has a mandate to direct and coordinate international health activities [83]. To do so, the organization is active in six main policy areas: health systems; promoting health through the life-course; non-communicable diseases; communicable diseases; corporate services; and preparedness, surveillance, and response [83]. The WHO’s highest decision-making body is the World Health Assembly, which meets annually to determine the organization’s policies on a one-country-one-vote basis. The Assembly appoints a Director-General every 5 years, supervises the WHO’s finances, including review and approval of its budget, and also elects 34 member state representatives to the WHO’s Executive Board for 3-year terms. The Executive Board nominates the Director-General, sets the agenda for the Assembly’s annual meeting, and sends resolutions and reports to the Assembly for consideration. Its main functions are to implement, advise, and “generally facilitate” the work of the Assembly. The Assembly and Executive Board are served by the WHO Secretariat, consisting of approximately 8000 experts and support staff located at the headquarters in Geneva and in the WHO’s six regional offices [84]. The WHO Regional Office for Europe (EURO) serves 53 member states—reaching far beyond the EU alone—and has established strategic partnerships with other European regional organizations, including the EU and the Organisation for Economic Co-operation and Development [85]. Each year, the WHO EURO holds a Regional Committee meeting, where member states vote on a one-country-one-vote basis to formulate policy, fulfill oversight functions, and approve the WHO EURO’s budget [86].

The WHO is financed by assessed and voluntary contributions, primarily from its member states and also from private sources. Assessed contributions are essentially membership dues required from all WHO member states, and they are adjusted according to the wealth and population size [87]. In the 2014–2015 biennial, the organization proposed a program budget of $3.98 billion [88]. In 2014, the WHO’s program budget was $2.5 billion, with 20 % ($492 million) of its funding coming from assessed contributions and 80 % ($2 billion) coming from voluntary contributions [89]. Voluntary contributions come primarily from member states (51 %) and also from the United Nations and other intergovernmental organizations (26 %), private foundations, such as the Bill and Melinda Gates Foundation (14 %), non-governmental organizations and institutions (7 %), and the remainder from the private sector (2 %) [89]. Most voluntary contributions are earmarked for the provision of health services in line with other development projects or in response to humanitarian crises [88].

#### Migrant health-related activities in Europe

The WHO EURO uses its convening power to hold high-level meetings and to create regional health policy frameworks (such as Health 2020) with which to coordinate and harmonize member state responses to migration [90]. It also collects and manages information on migrant health, which it uses to provide technical assistance to member states in the form of health-system assessments and policy advice [91]; on occasion, it also directly supports health interventions. The WHO EURO coordinates its work on migration and health through the Public Health Aspects of Migration in Europe (PHAME) project, which was established in 2012 with the support of the Italian Government [92]. It helps member states identify and fill gaps in health service delivery for migrants and provides policy recommendations on how best to enhance preparedness and to respond to migrant influxes [92]. Before this in 2011, the WHO EURO supported a conference and task force that recommended a minimum package of cross-border control and care for tuberculosis, a disease that disproportionately affects migrant populations in Europe [39]. To facilitate knowledge exchange between researchers and policymakers, the WHO EURO hosts the European Observatory on Health Systems and Policies, which has actively pursued a research agenda on migrant health issues over the years [93].

The WHO EURO has taken a number of actions to address the challenges facing health systems across Europe as a result of the recent influx of refugees and asylum seekers [94]. In August 2015, the WHO established an interdivisional Migration and Health Task Force in Europe charged with responding to the increase in calls for assistance from member states. At the country level, the WHO actions to respond to the migrant and refugee crisis include supporting the assessment of refugees’ needs, strengthening the capacity of medical staff, providing training to health care workers at points of entry, providing technical and financial assistance to outbreak response and immunization campaigns, procuring medical equipment and drugs, and disseminating health information to refugees [95]. The WHO EURO has also released a number of Health Evidence Network reports on migrant health focused specifically on the barriers to health care access for different migrant populations including refugees and asylum seekers [96], labor migrants [97], and undocumented migrants [98], with additional reports to come on mental health and maternal health for migrants and refugees [91], and on how the conceptual and legal definitions of the term “migrant” affect health care access and delivery to migrant populations. Lastly, the WHO EURO convened a high-level meeting on refugee and migrant health in Europe in November 2015 [91]. After this meeting, countries in the WHO European Region have agreed to prepare a common framework “for coordinated collaboration and action on refugee and migrant health” [99] among the WHO EURO partners, including other UN agencies, the EC, the IOM, and other national and international organizations [99].

### Médecins du Monde (MdM)

#### Background and governance

Founded in France in 1980 by a group of physicians, MdM quickly expanded its activities in both developing countries—often in crisis situations—and in Europe. The organization’s activities are guided by its principles of “respect for human dignity, information and protection of the person,” non-discrimination for those in need of medical care, and organizational independence [100]. Conceived as having international reach from the onset, today, the organization operates both international interventions via ad hoc missions and more stable programs in 15 “network countries” (ten of which are in Europe). The primary role of the latter is to provide access to health care services to vulnerable groups, including migrants [101].

The organization’s highest decision-making body is the general assembly, composed of over 1000 individuals meeting once per year and electing the members of the Board of Directors [102]. The latter numbers 12 members—at present, all doctors—with 3-year terms, who meet monthly to manage the organization [102]. The Board is supported by a permanent bureaucracy headed by a Director General, as well as advisory groups and a donor committee that gives voice to donors in the Board and general assembly.

The financing of MdM emanates primarily from public donations (52 % in 2014) and public institutional grants (42 %), with the remainder stemming from private sector grants and other sources [103]. The public institutional grants of the organization originated primarily from the European Union or national and international organizations (including the WHO—see above). The organization identifies a range of other INGOs (like the OSF—see below), national NGOs or public benefit foundations (e.g., Elton John Foundation or L’Oreal Foundation), private sector companies, and banks (e.g., Société Générale, Renault, American Express Japan) as private partners. Overall, the 2014 MdM international network budget amounted to €135 million, of which €77.9 represented the budget of MdM France, where MdM headquarters are located [103]. These funds were spent on social interventions (81 %), fundraising programs (13 %), and operating expenses (6 %). As of 2014, MdM’s personnel reached 4000, more than half of which were volunteers [103].

#### Migrant health-related activities in Europe

Issues related to migration and migrant health are among MdM’s key priorities, where its objectives are both to provide access to services and to “bear witness” to migrant’s experiences and challenges during their journey. To meet this objective, MdM has developed a range of interventions, often through the direct provision of health care services via ad hoc MdM-operated facilities. For instance, in 2013, the organization provided medical and social consultations to 16,881 patients in eight European countries [104], and in 2014, to 22,171 patients in nine European countries [105]. The majority of these patients were non-EU migrants (78 % in 2014) [105].

In particular, many of the MdM interventions target women’s and children’s health as well as infectious disease prevention. For instance, MdM centers in France provided free-of-charge HIV and hepatitis B and C screening for people living in extreme poverty, over 92 % of which were migrants [43, 44]. Similarly, in Greece, the organization operates four free-of-charge clinics around the country, as well as harm reduction programs for drug users in Athens [106]. Further, the Greek chapter provides medical and social services to migrants in Western Greece [107] and the islands of Lesbos and Chios—both key points in refugees’ migratory routes into Europe [106]. Services provided include infectious disease prevention, mental health support, and referrals to the national health system [106].

Further to the direct-provision services, MdM also collects, analyzes, and publishes data. These activities provide an evidence base for advocacy and outreach activities, many of which draw attention to the denial of migrants’ right to health care. The organization established an Observatory on Access to Healthcare in 2004, which publishes reports on MdM services and the health problems faced by the patients that visit the organization’s clinics [105]. Pursuing an agenda-setting role, the organization established a “European network to reduce vulnerabilities in health” that includes the European members of the MdM network and NGOs from European countries. The Network seeks to improve the capacities of its members on issues of health service provision, data collection, and outreach [108].

### Médecins Sans Frontières (MSF)

#### Background and governance

Founded in France in 1971 by a group of doctors and journalists, MSF is a private international association composed primarily of health professionals [109, 110]. The organization was created to advance equitable access to health care around the world [109], and its charter states explicitly that it “observes neutrality and impartiality in the name of universal medical ethics and the right to humanitarian assistance” [110]. In 2014, MSF had 384 projects in operation in 63 countries, ranging from the treatment of injuries and disease to the provision of humanitarian aid [111].

The organization’s highest decision-making body is MSF’s International General Assembly (IGA), which elects the International President and delegates management and oversight to MSF’s International Board. The latter acts on behalf of the IGA and meets about eight times per year to fulfill its duties, which include ensuring the implementation of IGA decisions, appointing a Secretary General to serve as the executive head of MSF International in Geneva, and overseeing the performance of MSF’s executive body, which is responsible for daily operations [112]. The Board consists of 12 members, plus a non-voting Treasurer, and is chaired by the International President [112, 113]. The Board’s membership consists of one representative from each of MSF’s five operational centers, six members who are elected by the IGA, and the International President.

MSF is financed primarily by private donations (89 % in 2014) and public institutions (9 %), with the remainder stemming from other sources. The majority of MSF’s private donations (86 %) were from individuals, with the rest coming from private institutions including companies, trusts, and foundations [114]. MSF funding from public institutions came primarily from Europe (84 %) including contributions from individual EU member states and EU institutions—including the EC’s Humanitarian Aid and Civil Protection DG (see above)—with the remainder coming from governmental institutions outside of Europe [114]. Overall, MSF spent €1.1 billion in 2014, of which 80 % was spent on humanitarian activities and 20 % on management and fundraising activities [115]. As of 2014, MSF has a staff of over 36,000, including local (85 %), international (8 %), and headquartered staff (7 %) [115]. All field staff (both local and international) are compensated but not employed; together, their transport, room and board, health insurance, emergency evacuation insurance, and per diem account for just over half of MSF’s annual expenses [115, 116].

#### Migrant health-related activities in Europe

In line with its mission, MSF’s primary goal is to prevent loss of life, and it works to serve all populations in need of health care. For MSF, health care includes both physical and mental health services, which it provides to migrants in both transit and receiving countries and also en route to their destination [45]. In 2014, MSF provides services in the following transit and receiving countries of European migrants: Tunisia, Italy, Greece, Serbia, Croatia, Slovenia, and France [117]. MSF also advocates for better coordinating the services it provides with local authorities of the receiving countries in which they work in order to improve health care in the medium and the long term for migrants [46].

In 2002, MSF began working in Lampedusa reception center in Italy to provide medical care to asylum seekers and has continued to support European migrants since [118, 119]. 2015 was the first year in which MSF has operated search and rescue ships in the Mediterranean for migrants attempting to cross the sea [120]. By the end of the year, MSF ships had rescued over 18,000 people [117]. In addition to health care, MSF provides training to local fisherman on how to assist in search and rescue missions should they come across a boat in need of help and also training in dead body management, as fishermen are often the first to encounter migrant vessels [117]. Large numbers of migrants display signs of having experienced physical and psychological violence; MSF focuses on providing not only physical but also mental health support to those who have experienced such trauma [117]. When necessary, MSF also provides shelter, water, sanitation, and other essentials to prevent the spread of disease in arrival locations, particularly where there is a lack of reception facilities or in the event that existing facilities are overwhelmed [117]. For example, on Greece’s Leros island, MSF carried out 7113 health consultations and distributed 12,210 relief items from mid-March to the end of October 2015 [117]. MSF also works with MdM to support migrant health in Calais, a crossing point from France to the UK [117]. There, MSF provides mobile clinic services, and it works to address the shelter, water, and sanitation needs of migrants, complementing the medical consultations and physiotherapy provided by MdM.

In total, MSF has provided nearly 100,000 medical consultations to refugees and migrants traveling to Europe and have treated 12,214 patients for trauma-related conditions. They spent an estimated €31.5 million and required 534 staff members to respond to the migrant crisis in 2015 [121]. In January 2016, MSF released a report criticizing the EU’s response to the migrant crisis. The report recommends that the EU and its member states provide safe and legal channels for people seeking asylum, to undertake search and rescue operation at sea, and to improve their approach to reception, with a focus on meeting both physical and mental health needs [121].

### Open Society Foundations (OSF)

#### Background and governance

Founded in 1979 in the USA by financier George Soros, OSF’s objective is to contribute to building “vibrant and tolerant societies whose governments are accountable and open to the participation of all people” [122]. In practice, this objective translated into a multitude of programs around the world. The organization maintains “core offices” in four countries and several “satellite offices” across geographical regions [123]. The organization is governed by a global board and a range of regional and thematic boards, overseeing areas such as public health or international migration [124].

In the past three decades, the organization spent over $31 billion in pursuit of its objectives, including $737 million on public health issues globally [125]. In 2016 alone, the budget amounts to $930.7 million, of which 58 % is devoted to grant-making programs [123]. Among these, the health and rights policy area absorbs $35.7 million (6.6 % of program expenditures) and $12.5 million (2.3 %) are devoted to migration; most funds in both policy areas are directed to programs in Europe [123].

#### Migrant health-related activities in Europe

OSF involvement in migrant health-related issues in Europe primarily operates through two key channels: grant-making and advocacy/evidence collection. For example, over the years, OSF has funded interventions by medical NGOs across Europe. In Italy, the organization financed an intervention by Italian NGO Doctors for Human Rights that provided medical and psychiatric services to asylum seekers [126].

Second, OSF supports a range of advocacy activities and related evidence gathering, mostly related to access to health care for disadvantaged social groups, including migrants. The organization is among the key funders of organizations active in this area, like the Platform of International Cooperation for Undocumented Migrants (PICUM) [127]. OSF also supports a range of policy initiatives in the fields of medicine policy, sex worker rights, and harm reduction which—indirectly—relate to migrant health issues.

## Conclusions

Our findings reveal that IGOs and INGOs are involved in both service provision and policy shaping and are interconnected via joint projects or cross-financing. States often request the expert policy advice of IGOs, especially in the case of emergencies or for the design of policy interventions. Further, states, IGOs, and INGOs occasionally contract other IGOs, INGOs, or domestic NGOs to deploy interventions. These linkages yield a complex web of relationships, which can vary depending on the country under consideration or the health policy issue in question (e.g., infectious disease control or mental health services).

Before discussing our findings, we note three limitations of our work. First, we have provided a bird’s eye view of the operations of six multifaceted international organizations; we did not set out to cover every activity of these organizations in every European country in which they operate. Further, the nature of this study limited us to considering the public-facing output of these organizations as expounded in factsheets, official reports, public statements, or other freely available information. This evidence is suggestive but not always representative of the range and impact of organizations’ activities [13]. In addition, other important IGOs and INGOs—like the UNHCR or the International Red Cross—also are involved in this policy area but their activities are not discussed here. Future research could expand the breadth and depth of our understanding of this policy field and could also utilize diverse methodological approaches, such as expert interviews with organizational stakeholders and archival research. Second, this review highlights the output of these organizations without reference to the desirability, effectiveness, or quality of these interventions. For example, several of the academic articles identified in our search for literature on this topic focused on aspects of migrant health that have not been adequately addressed, such as the unique needs of migrants who have also been victims of sexual violence [35, 37], the standardization of health monitoring across EU countries to ensure the availability of comparable data on migrant health [33], and the standardization of screening for infectious disease across EU member states [36]. Finally, the lack of standardized data collection and publication from INGOs on their interventions on migrant health in Europe prohibits systematic and comprehensive assessments of their work across their continent. The MdM reports [104, 105, 128] on access to health care by vulnerable groups in their facilities across Europe reflect current best practice and could be emulated by other actors in the field.

As suggested in earlier parts of this study, the six international organizations reviewed here interact in various ways with other IGOs, INGOs, national governments, and local NGOs. While the interactions are numerous, the relations between these groups of actors are not necessarily harmonious. For example, in response to the deal between the EU and Turkey aimed to curb the number of refugees reaching Greece, MSF announced its strong condemnation and refusal to “receive funding from institutions and governments whose policies do so much harm” [129]. Similarly, the provision of health services to migrants by various INGOs within European countries is itself an indication of inequities in health care access within European countries according to migrant status.

Our findings have important implications for policy. First, enhancing policy coherence and coordination among international organizations is critical for overcoming fragmentation, avoiding turf wars, and preventing the parallel provision of services—phenomenon that are likely to occur in the absence of a clear delineation of organizational responsibilities and amid competition to lead projects [130]. Establishing clear lines of authority and functional differentiation among IGOs is key for streamlining activities. In the case of INGOs, parallel provision of services often arises from a lack of coordination and also from attempts to fill gaps in service provision in areas where the state has retreated [131]. When addressing these problems, policymakers must weigh the merits of creating structures and systems that cater to the unique, and sometimes acute, health needs of migrants against the merits of including migrants in standard health and welfare services. Second, there appears to be a persistent gap between the degree to which international organizations aspire to improve migrant health and the resources they are able to mobilize in order to do so. While both IGOs and INGOs have financial limitations, IGOs do not have the flexibility to amend their mandates or goals, making it difficult for them to manage public expectations. In particular, the WHO’s heavy reliance on voluntary contributions, especially for activities falling outside of its biennial budget, severely limits its ability to respond to unexpected events, including the influx of migrants in Europe.

The role of international organizations in the field of migrant health will likely become more relevant in the years to come as a result of increasing migration flows. Future research can examine some central questions that will contribute to our understanding of how these organizations affect migrant health in Europe. First, since migrant health remains a national competency throughout Europe, the interaction between governments, IGOs, and INGOs requires more attention: How are policies developed in these areas, and how do the inputs of this diverse array of actors fit into the policymaking process? Under what conditions do governments decide to contract services to INGOs or seek the assistance of IGOs? Second, we still lack a comprehensive picture of the different sets of actors that migrants encounter during their migration journey: from the country of origin, through transit countries, to the country of destination. Across this route, migrants encounter a multitude of actors providing a range of services; a map of such services would aid effective policy design. Third, we know little about the effectiveness and quality of neither IGO nor INGO interventions in the field of migrant health; subsequent studies can shed light on these issues. Finally, the recent refugee crisis intersects with economic crises still affecting in a number of European countries, notably Southern Europe. Past studies have shown that economic crises adversely affect migrant health (including via restrictions to migrants’ eligibility to access health services) [131–139]; further evidence is necessary to document how these ailing economies are relying on IGOs or INGOs for the provision of services or policy support.

## Abbreviations

EC: European Commission
IOM: International Organization for Migration
MdM: Médecins du Monde
MSF: Médecins Sans Frontières
OSF: Open Society Foundations
UNHCR: United Nations High Commissioner for Refugees
WHO: World Health Organization
